# A Numerical Method Charactering the Electromechanical Properties of Particle Reinforced Composite Based on Statistics

**DOI:** 10.3390/polym10040426

**Published:** 2018-04-11

**Authors:** Mengzhou Chang, Zhenqing Wang

**Affiliations:** College of Aerospace and Civil Engineering, Harbin Engineering University, Harbin 150001, China; changmengzhou@hrbeu.edu.cn

**Keywords:** polymer composite, electromechanical property, modeling and simulation, particle, statistics

## Abstract

A novel model for a network of polymer chains is proposed considering the distribution of polymer chains inside the composite in this work. Some factors that influence the distribution of polymer chains are quantitatively investigated, such as external surface geometry, internal filler, and local deformation. Furthermore, the Maxwell stress induced by an electric field is characterized by the statistics of local charge density, as the basic analyzing electromechanical properties of materials. In particular, taking the non-uniform distribution of polymer chains into account, the electromechanical properties of two materials—VHB 4910 and CaCu_3_Ti_4_O_12_-polydimethylsiloxane (CCTO-PDMS)—are investigated to validate the applicability of the proposed model. The comparison between simulation results and experimental results from existing literature shows that the model was successfully employed to predict the electromechanical properties of polymer composites.

## 1. Introduction

Structures consisting of soft materials can undergo large deformation under the effects of external electric fields and force fields, and can be used for different applications such as actuators, artificial muscles, and energy harvesters [1,2]. The experimental investigations of planar actuators have shown that the Maxwell stress induced by the external electric field is generally proportional to the voltage value, and in inverse proportion to the thickness [3]. Methods to reduce the driving voltage (generally kV) have been investigated, including reducing thickness (thin membrane), expanding length (pre-stretch), and increasing dielectric permittivity (adding filler). However, the applications of soft materials are limited due to their failure mechanism both for pure mechanical (loss of tension, rupture by stretch and aging) and electromechanical loading (dielectric breakdown, wrinkling/buckling, and loss and instability) [4]. Therefore, there have been many attempts to develop polymer–filler composites or new structures to overcome the drawbacks mentioned above [5,6].

As for composites with different geometries or conductive fillers, the material properties are quite complex and related to the microstructures [7,8]. The morphology examined by scanning electron microscopy (SEM) indicates that around particles or near surfaces, the polymer chains will re-distribute according to the manufacture process and the interaction between the fillers and the matrix [9,10]. Finite element method at microscale, principle of energy conservation at macroscale, and modification of parameters are generally used to solve this problem in these composites [11,12,13].

In recent years, conductive fillers have been added to polymer-based composites to develop high-performance dielectric polymers (high dielectric permittivity and low loss tangent), such as carbon nanotubes [14,15], graphite [16,17], metal particles [18,19], and conductive oxides [20,21,22]. Generally, higher dielectric permittivities can be achieved in composites by adding these particles, however, unexpected loss tangents are also found, especially under high temperature and frequency. Among those particles, CaCu_3_Ti_4_O_12_ (CCTO) shows a high dielectric permittivity which is almost unchanging over a range of temperature and frequency greater than 10^4^ [23,24,25].

The macro-mechanical properties should originate from the microstructure of polymers, including spatial distribution, interfacial reaction, and chemical modification [26,27]. Based on the analysis of microstructure [28], several multi-scale models have been proposed, such as the three-chain model, eight-chain model, and tetrahedral model [29,30,31]. Beyond that, hyperelastic models aiming to capture the limiting stretch of polymer chains are always used to describe the mechanical behavior of soft polymers [32,33]. A micro-mechanical model for finite rubber elasticity based on a non-affine network and topological constraints was proposed by Miehe and Göktepe [34]. The finite rubber viscoelasticity and anisotropic Mullins-type damage is further discussed in their follow-up articles [35,36]. By studying experimental data and the microstructure of carbon black-filled rubbers, the anisotropic Mullins effect is investigated by Dargazany and Itskov [37]. In this work, a damage polymer–filler network is introduced to qualitatively illustrate chain sliding on or debonding from aggregates. The model is further developed in their work: chain length statistics are incorporated into full network rubber [38]; a closed form of the Rayleigh exact distribution function for non-Gaussian chains is considered [39]; a cross link-particle (CP) network is added to the elastic rubber (cross link- cross link; CC) and polymer-filler (particle-paritcle; PP) network [40]. Moreover, several studies in the literature focusing on the electromechanical properties of polymers revealed the relationship between network structure and electromechanical loading/response [41,42].

The foundation of the models mentioned above is the hypothesis of the random distribution of the polymer chain. The objective of this work is to develop a theoretical model to quantify the structure–function relationship by modifying the distribution of the polymer chains. The distribution of the polymer chain is determined by the geometry characteristic and the interaction between matrix and fillers. This work could shed some light on the development of polymer-based composites.

## 2. Improved Visco-Hyperelastic Model

### 2.1. Strain Energy Potential and Viscoelasticity

**Figure 1:**
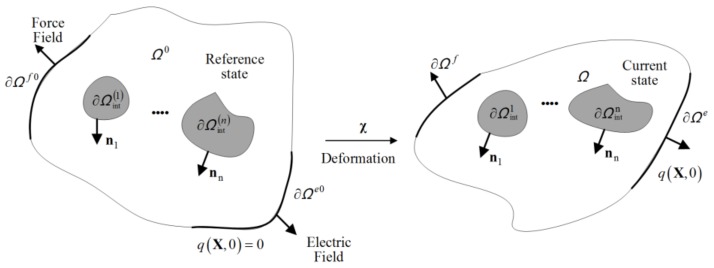
Illustration of the system in reference state *t* = 0 and current state *t*.

A system $\Omega^{0}$ with internal structures $\partial \Omega_{\mathrm{int}}^{\left(1\right)}$, $\partial \Omega_{\mathrm{int}}^{\left(2\right)}\cdots \partial \Omega_{\mathrm{int}}^{\left(n\right)}$ (with normal vector $n_{1},\;n_{2},\;\cdots ,\;n_{n}$) and outer space $\Omega_{\mathrm{sur}}^{0}=$ 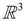
$/\Omega^{0}$ is introduced to illustrate the model. At the reference state *t* = 0, the undeformed stress-free configuration will be maintained. At time *t*, force field and electric field are applied on the outer space $\partial \Omega_{ext}^{f0}$ and $\partial \Omega_{ext}^{e0}$, respectively. Meanwhile, the system has changed to current configuration Ω. The deformation is described by function χ, which maps a reference particle X in $\Omega^{0}$ to its deformed position x=χ(X) in Ω. The associated deformation gradient will be denoted by F=∂χ/∂X, and J=det(F) identifies its determinant, as shown in Figure 1.

In arbitrary subregion $\Omega_{\mathrm{sub}}\subset \Omega^{0}$, the integral of the flux density over a closed surface equals the charge enclosed based on Gauss’s law:

(1) $$\oint_{\Omega_{\mathrm{sub}}}\rho d\Omega =\sum Q,$$

where ρ and Q are the flux density and charge, respectively. The constitutive equations for current density **I**, electric field **E**, and charge **Q** are expressed as:

(2) ∇I=0,

(3) $$\nabla E=\frac{\rho }{\varepsilon \varepsilon_{0}},$$

(4) I=μρE,

(5) E=−∇V,

where μ is the electric mobility, **V** is the applied voltage, ε and $\varepsilon_{0}$ are the dielectric permittivity for material and free space, respectively. We should note that Equations (2)–(5) are suitable for materials that have relatively stable performances. However, dielectric permittivities of most of soft materials are reported as a function of the deformation and frequency *f*, which can be expressed as ε=ε(χ,f).

A surface charge with density ρ is formed under the effect of voltage **V**. Thus, the Maxwell stress is induced by *q* and the electric field $P_{e}\left(q,t\right)=Q\cdot E$ [43]. With force **P**_f_, a new shape is formed $\Omega^{0}\left(t+\Delta t\right)$; this is beyond the explanation of pure force working on $\partial \Omega^{f}$. Similarly, a new surface charge density is formed:

(6) $$\rho \left(t\right)\overset{\;E\;}{\underset{\;P_{f}\;}{\to }}\Omega \left(t\right)\overset{\;E\;}{\underset{\;P_{f}\;}{\to }}\rho \left(t+\Delta t\right).$$

The local electric breakdown will happen when the electric field of two points $A^{\prime }\;\mathrm{and}\;B^{\prime }\subset \Omega^{0}$ is larger than the dielectric breakdown strength $E_{bd}$ [44]:

(7) $$E\left(A^{\prime }B^{\prime }\right)\geq E_{bd}.$$

In order to ensure global electric breakdown, Equation (7) is extended to two points on the outer surface A and B $\subset \partial \Omega^{0}$: the general electric breakdown of the system happened if a line with two ends A and B across the body are found.

### 2.2. Distribution of Polymer Chain

**Figure 2:**
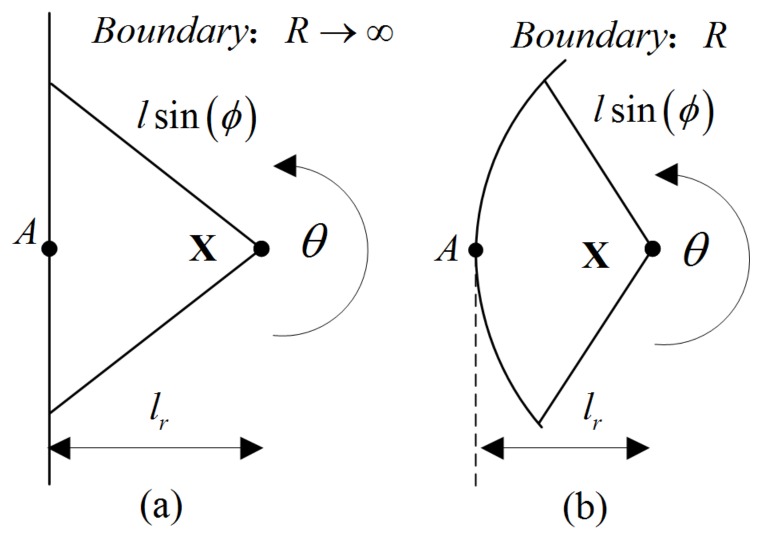
Effect of surface on the distribution of polymer chains on a plane: (**a**) R→∞; (**b**) R.

In this section, the effective polymer chain length *L* = *nl* (*L* is the total length of a polymer chain; *n* is the segment number; *l* is the length of a single segment) is introduced to investigate the distribution of polymer chains. We start by considering the random distribution, which means the polymer chain is inside the system and far from any boundaries (both inside and outside). In this situation, a spherical coordinate system (r,θ,ϕ) is used for a better description, and the possible rotation angle of a polymer chain around point **X** can be expressed as:

(8) 0≤θ(X)<2π,

(9) 0≤ϕ(X)≤π,

where θ and ϕ are the space rotation angles. Space rotation angle will be restricted when the point is near boundary. As shown in Figure 2, a plane with X is intended to illustrate the restricted behavior of a surface with different radius of curvature R (*r*, 0≤θ(X)<π, ϕ). Particularly, the space rotation angle of point X can be obtained:

(10) $$\begin{array}{c} R\to \infty :\arccos\left(\frac{l_{r}}{l\sin\left(\phi \right)}\right)\leq \theta \left(X\right)\leq 2\pi -\arccos\left(\frac{l_{r}}{l\sin\left(\phi \right)}\right)\\ R:\;\pi -\arccos\left(\frac{R^{2}-l^{2}\sin^{2}\left(\phi \right)-{\left(R-l_{r}\right)}^{2}}{2l\sin\left(\phi \right)\left(R-l_{r}\right)}\right)\leq \\ \theta \left(X\right)\leq \pi +\arccos\left(\frac{R^{2}-l^{2}\sin^{2}\left(\phi \right)-{\left(R-l_{r}\right)}^{2}}{2l\sin\left(\phi \right)\left(R-l_{r}\right)}\right) \end{array},$$

where $l_{r}$ is the distance from point X to a point A on the boundary.

The probability of random walking in one dimension without restriction can be expressed as a Gaussian distribution:

(11) $$p\left(x,n\right)=C_{n}^{0.5\left(n+m\right)}{\left(0.5\right)}^{n},\;0\leq m\leq n,$$

where *x* = *ml* is the walking distance and *m* is the testing step measured from the origin (−n≤m≤n).

**Figure 3:**
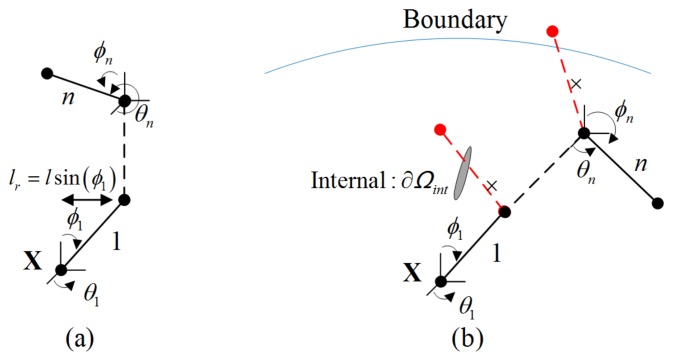
The distribution of polymer chains: (**a**) random distribution; (**b**) restricted distribution.

The random walking in three dimensional space can be described as: *n* set of rotational angles θ and ϕ have arbitrary value ($0\leq \theta_{i}<2\pi$ and $0\leq \phi_{i}\leq \pi$, *i* = 1, 2, …, *n*) as shown in Figure 3. In contrast, the values of θ and ϕ under the restricted condition can be expressed by Equation (10). It is interesting to note that internal structures have different effects on the distribution of the polymer chains according to the interaction of different materials and the manufacturing operation: first, with gas-like material in an internal surface $\partial \Omega_{\mathrm{int}}^{0}$, the polymer chain will automatically avoid the region, resulting in a conditional distribution of polymer chains in this region; second, with particle reinforced in an internal surface, the polymer chains show cross-linking characteristic around this region, meaning that several chains will share the same point. The length of polymer chains will be changed due to this reinforcement. Generally, we assume that $n_{cro}$ chains are walking through this region, and $n_{com}$ segments are cross-linked around the particle; thus, the modified segment number of the chain can be expressed as:

(12) $$n_{in}=\frac{n_{cro}\times l+n_{com}}{n_{cro}}=kn,$$

where $n_{in}$ is the internal segment number of the reinforced chain.

**Figure 4:**
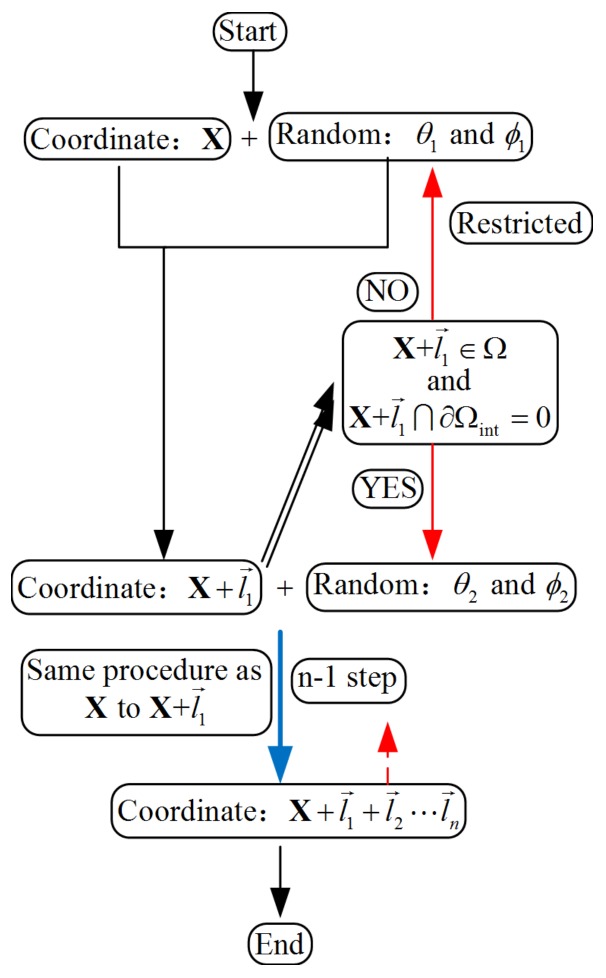
The numerical method used to calculate the probability distribution.

In Equation (12), it is obvious that the segment number is larger than that of random distribution. For the convenience of simulation, it is suggested that a parameter *k* (*k* > 1) should be used in this situation. The details of the numerical method are shown in Figure 4. According to Figure 4, *n* set of $\theta_{i}$ and $\phi_{i}$ (*i* = 1, 2, …, *n*) will first be automatically generated. Combining the coordinate of X, the coordinate of the point $X+{\vec{l}}_{1}$ will be obtained. Following the same procedure, the new coordinate of point $X+{\vec{l}}_{1}+{\vec{l}}_{2}$ will be obtained if the coordinate ($X+{\vec{l}}_{1}$) meets the condition of constraint as shown in Figure 4. Otherwise, the values of $\theta_{1}$ and $\phi_{1}$ should be re-calculated according to Equation (10). Repeating the same step, the coordinates of the *n* points can be obtained.

Considering the restricted behavior, the probability tested from point X to another point $X^{\prime }$ is obtained as:

(13) $$p\left(X,X^{\prime }\right)=\frac{X^{\prime }-X}{\sum_{i=1}^{n}i{\vec{l}}_{i}}.$$

### 2.3. The Mechanical Model of Soft Materials

The relationship between stress σ and strain ε=λ−1 can be summarized as follows [45,46]:

(14) $$\sum_{k=0}^{m}p_{k}\frac{d^{k}\sigma }{dt^{k}}=\sum_{k=0}^{n}q_{k}\frac{d^{k}\varepsilon }{dt^{k}},$$

where p and q are the material parameters. Equation (14) is the general expression for the modified Kelvin–Voigt model. Applying the hydrostatic pressure $p_{hy}$ into Equation (14), we can rewrite the stress–strain relationship as:

(15) $$\sum_{k=0}^{m}p_{k}\frac{d^{k}\left(\sigma_{ij}+P_{\mathrm{hy}}\delta_{\mathrm{ij}}\right)}{dt^{k}}=\sum_{k=0}^{n}q_{k}\frac{d^{k}\varepsilon_{ij}}{dt^{k}},\;i=x,y\;\mathrm{and}\;z,$$

where $\delta_{ij}$ is the Kronecker delta ($\delta_{ij}$ = 1 if *i* = *j*, and $\delta_{ij}$ = 0 otherwise). In Equation (15), only normal stress is considered (shear stress can be ignored) as the main mode; shear stress is inappropriate for soft materials.

Many studies have attempted to separate the time-dependent behavior from mechanical models by introducing a time function, as can be seen in [46,47]. Similarly, the equivalent modulus (a function of time and strain) can be used for the characterization of a viscoelastic material:

(16) $$\sigma +P_{\mathrm{hy}}I=E_{equ}\varepsilon ,$$

where $E_{equ}$ denotes the equivalent elastic modulus and I is the unit tensor.

When we consider *np* models connected in parallel, the relationship between stress and strain of the point X integrating probability can be expressed as:

(17) $$\begin{array}{c} \sigma +P_{hy}I=nPE_{equ}\varepsilon \\ \mathrm{or}\;\sigma +P_{hy}I=n_{in}PE_{equ}\varepsilon \end{array},$$

where **P** is the matrix expression of probability as shown in Equation (13).

### 2.4. Re-Distribution of Charge on the Surface

**Figure 5:**
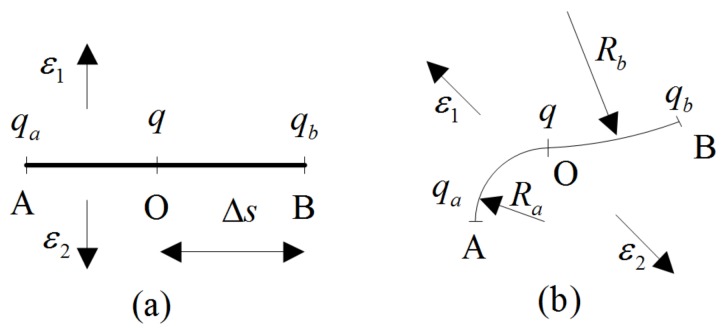
Distribution of charges on the outer surface of material: (**a**) homogeneous; (**b**) inhomogeneous.

In this section, two distribution laws of the charges on the surface of a material are investigated. Firstly, the homogeneous distribution which is usually used in the planar actuator is shown in Figure 5a. Three points O, A, and B with distance Δs are chosen to study the distribution law. Two dielectric permittivities $\varepsilon_{1}$ and $\varepsilon_{2}$ are used to characterize the dielectric property inside and outside the material, respectively. The charges on three points at homogeneous state are the same:

(18) $$q=q_{a}=q_{b}.$$

Secondly, an assumption is made over a small time: the charge cannot move from a point to another point along a smooth surface, as shown in Figure 5b. Taking point O as an example, the components of force along the tangential direction ($e_{2}$) and the vertical direction ($e_{3}$) are as follows:

(19a) $$\begin{array}{ll} F\left(e_{2}\right)= & \frac{qq_{a}}{4\pi \varepsilon_{0}\varepsilon_{2}r_{a}^{2}}e_{2}-\frac{qq_{b}}{4\pi \varepsilon_{0}\varepsilon_{1}r_{b}^{2}}e_{2}\\ & =\left[\frac{qq_{a}\cos\left(0.5\theta_{a}\right)}{4\pi \varepsilon_{0}\varepsilon_{2}{\left(2R_{a}\sin\left(0.5\theta_{a}\right)\right)}^{2}}-\frac{qq_{b}\cos\left(0.5\theta_{b}\right)}{4\pi \varepsilon_{0}\varepsilon_{1}{\left(2R_{b}\sin\left(0.5\theta_{b}\right)\right)}^{2}}\right]e_{2}\\ & =0 \end{array},$$

(19b) $$\begin{array}{ll} F\left(e_{3}\right)= & \frac{qq_{a}}{4\pi \varepsilon_{0}\varepsilon_{2}r_{a}^{2}}e_{3}-\frac{qq_{b}}{4\pi \varepsilon_{0}\varepsilon_{1}r_{b}^{2}}e_{3}\\ & =\left[\frac{qq_{a}\sin\left(0.5\theta_{a}\right)}{4\pi \varepsilon_{0}\varepsilon_{2}{\left(2R_{a}\sin\left(0.5\theta_{a}\right)\right)}^{2}}-\frac{qq_{b}\sin\left(0.5\theta_{b}\right)}{4\pi \varepsilon_{0}\varepsilon_{1}{\left(2R_{b}\sin\left(0.5\theta_{b}\right)\right)}^{2}}\right]e_{3} \end{array},$$

where $\theta_{a}$ and $\theta_{b}$ are the angles, $\Delta s=R_{a}\theta_{a}=R_{b}\theta_{b}$. The deformation is changing due to force $F\left(e_{3}\right)$ and force field, which will result in charge redistribution. It is obvious that the ratio $q_{a}/q_{b}$ is a function of $\Delta s,R_{a}\;\mathrm{and}\;R_{b}$ (or ∂Ω) in a small area. When Equation (19) is extended to the general situation of the whole material, the following expression can be obtained:

(20) q=q(∂Ω,t).

Along with Equations (1)–(6), the charge redistribution behavior can be obtained at any time.

## 3. The Effect of External Structures on the Electromechanical Properties

### 3.1. Determination of the Material Parameters

**Figure 6:**
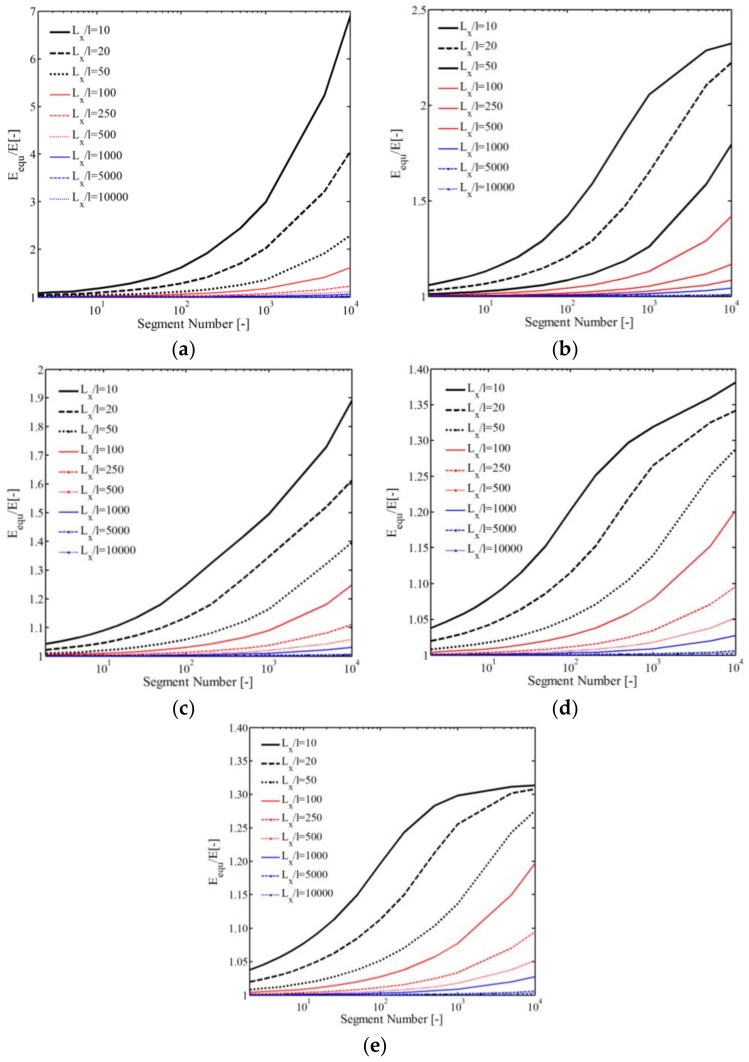
Effect of geometric effect on the equivalent elastic modulus: (**a**) $L_{y}/L_{x}$ = 1; (**b**) $L_{y}/L_{x}$ = 2; (**c**) $L_{y}/L_{x}$ = 10; (**d**) $L_{y}/L_{x}$ = 500; (**e**) $L_{y}/L_{x}$ = 10,000.

A sample with dimension $L_{x}\times L_{y}\times L_{z}$ ($L_{z}\gg L_{x}$, $L_{z}\gg L_{y}$) and loading in *z*-direction is taken to illustrate the influence of *l* and *n* on the mechanical properties. As shown in Figure 6, the equivalent elastic modulus $E_{equ}$ is increased with increasing segment number and decreasing with $L_{x}/l$. Considering $L_{x}=L_{y}$, a large ratio is found $E_{equ}/E\approx 7$ under condition *n* = 10,000 and $L_{x}/l$ = 10 (*E* is the elastic modulus equaling random distribution), as shown in Figure 6a. However, considering a dimension $L_{y}/L_{x}\geq 1$, the ratio is much smaller than that of $L_{x}=L_{y}$—especially $L_{y}/L_{x}$ = 10,000, as shown in Figure 6b–e.

### 3.2. Simulation of Rectangle Sample

**Figure 7:**
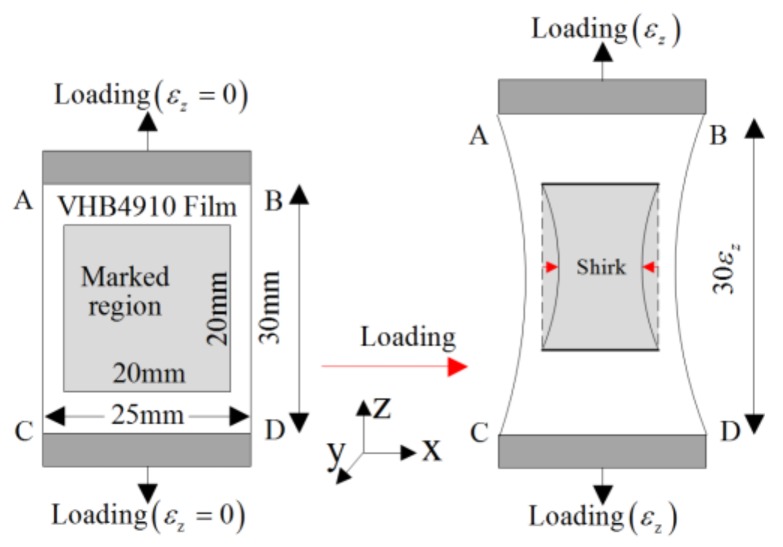
Illustration of VHB 4910 specimen under tension test [48].

As an example, tensile tests of VHB 4910 (Acrylic, 3M Company, USA) specimens were conducted. The specimen (25 mm × 1 mm × 30 mm) was clamped between the grips, then the specimen was loaded at a given rate. A rectangular region (20 mm × 20 mm) was marked in the center region of the sample. By doing so, the deformation mechanism was investigated, as shown in Figure 7.

**Figure 8:**
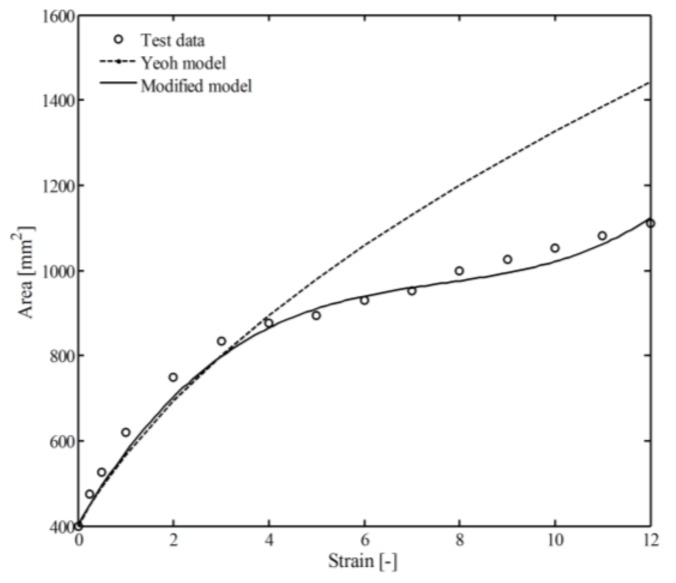
Relationship between the marked area and tensile strain [48].

The area of the marked region was recorded during the loading process and compared with the simulation results of the Yeoh model and the modified model, as shown in Figure 8. During the simulation process using the Yeoh model, the material was treated as homogeneous isotropic, the parameters and more details can be found in Reference [47] and in our previous work [49]. The minimum values of *l* and *n* were 0.08 mm and 15 for the modified model. The deformation of the sample can be summarized as: when strain ranged from 0 to 4, a homogeneous deformation could be observed; however, when strain ranged from 4 to 12, the deformation was affected by shrinking behavior of the boundary. As can be seen from Figure 8, the comparison between the simulation results of the modified and the experimental data shows good agreement with relative error 5.2% (28% for Yeoh model).

**Figure 9:**
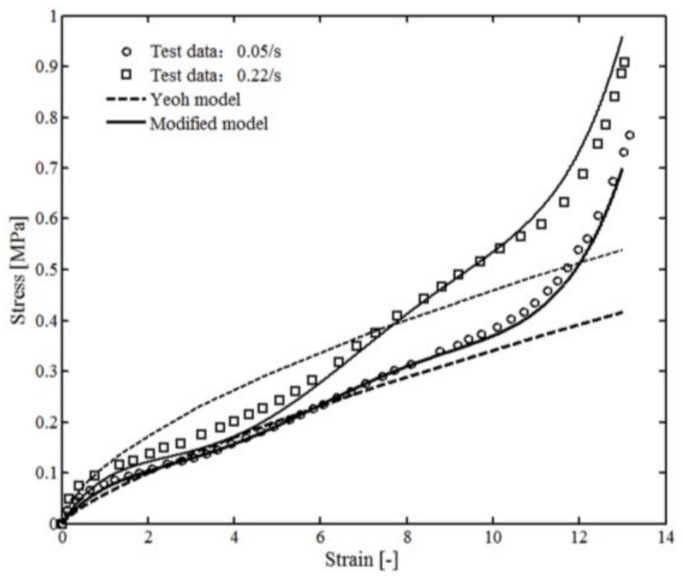
Relationship between stress and strain under different loading rates [48].

The stress–strain curves of VHB 4910 specimens under different loading speeds (0.05/s and 0.22/s) are shown in Figure 9. It was observed that VHB 4910 demonstrated typical elastomeric tensile behavior, which means a greater stress could be observed under larger strain or larger strain rate. In a state of small strain (ε < 4), both Yeoh model and the modified model were valid for the nonlinear mechanical behavior of VHB 4910. However, in a state of large strain state (ε > 4) only the modified model showed a promising result (ignoring the difference near the failure strain, ε≈ 13).

The difference of the two models can be explained as follows: the middle part of the specimen was shrinking during the loading process. This part reduced quickly using the modified model compared with the Yeoh model (especially for larger strain ε>4). Additionally, the equivalent elastic modulus calculated using the modified model (near the boundary region) became much larger compared with the Yeoh model (along the *z*-direction). Because of this, the material can be regarded as nonhomogeneous, which can further affect the deformation of the material.

### 3.3. Simulation of Circular Dielectric Elastomer

**Figure 10:**
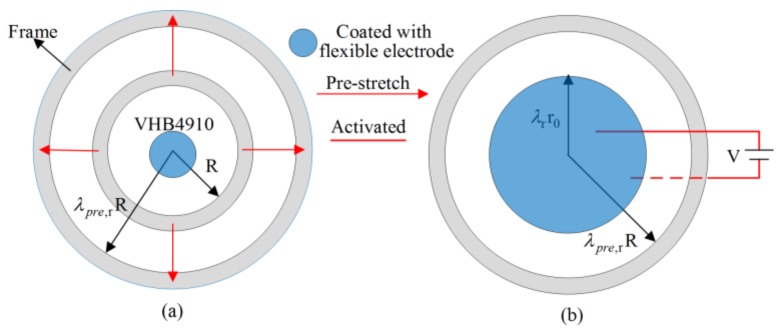
Working principle of a circular dielectric elastomer actuator [47]: (**a**) Pre-stretched state; (**b**) Activated state.

A circular elastomer membrane with radius R was fixed on a movable frame and coated with flexible electrode on both sides (radius $r_{0}$ = 7.5 mm) in the reference state. Then, the membrane was pre-stretched to a level $\lambda_{pre,r}R$ = 75 mm in the pre-stretched state, as shown in Figure 10a. In the activated state, the radius of active area changed into $\lambda_{r}r_{0}$ by applying an external voltage V on the active area, as shown in Figure 10b.

**Figure 11:**
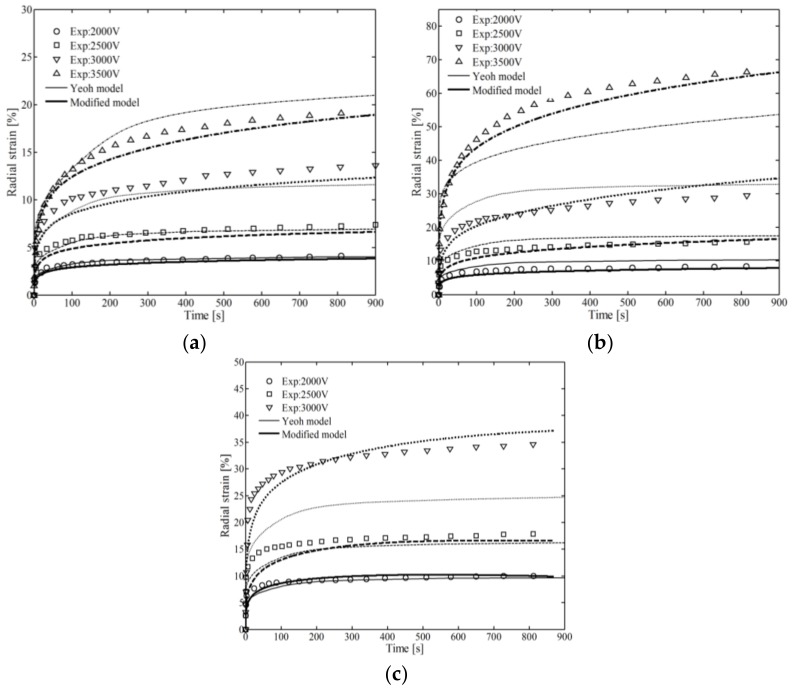
Fitting the improved model and Yeoh model to the circular dielectric elastomers: (**a**) $\lambda_{pre,r}$ = 3; (**b**) $\lambda_{pre,r}$ = 4; (**c**) $\lambda_{pre,r}$ = 5.

The relationships between radial strain of active area ($\lambda_{r}$ − 1) and time under different pre-stretched ratio $\lambda_{pre,r}$ and applied voltage V are plotted in Figure 11.

From Figure 11, some conclusions can be drawn as follows. Subjected to the same voltage, the radial strain is increased with the pre-stretched level. When the pre-stretched strain was held constant, the radial strain increased with increasing voltage. The radial strain changed temporality and quickly when at time *t* < 300 s, then remained stable over a longer period, *t* >> 300 s. The relative errors of the Yeoh model were 18% and 21% for test $\lambda_{pre,r}$ = 4, V = 3500 V and test $\lambda_{pre,r}$ = 5, V = 3000 V, respectively, as shown in Figure 11b,c. However, the relative errors of the modified model were 4.6% and 6.2%, which are much more acceptable.

**Figure 12:**
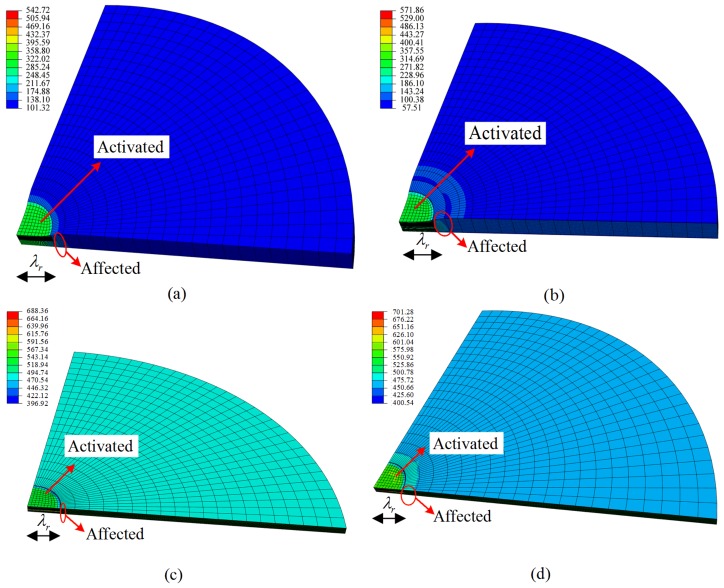
Simulation results of quarter model of the circular dielectric elastomer at time *t* = 300 s: (**a**) Yeoh model $\lambda_{pre,r}$ = 3 and V = 2000 V; (**b**) Modified model $\lambda_{pre,r}$ = 3 and V = 2000 V; (**c**) Yeoh model $\lambda_{pre,r}$ = 5 and V = 3000 V; (**d**) Modified model $\lambda_{pre,r}$ = 5 and V = 3000 V.

As for lower pre-stretched level and voltage, the simulation results of the two models were close, as can be seen from Figure 12a,b. However, at higher pre-stretched level and voltage, the boundary of the activated area extended with time more quickly using the modified model compared with the Yeoh model, as shown in Figure 12c,d.

## 4. The Effect of Internal Particles on the Electromechanical Properties

### 4.1. Determination of the Material Parameters: Local Density

**Figure 13:**
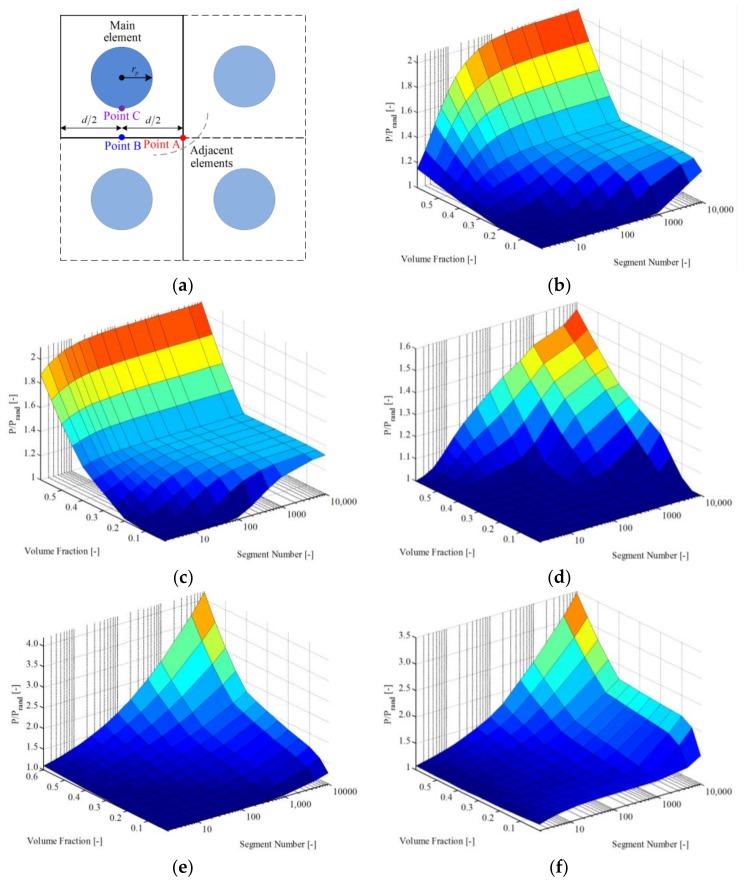
Density of polymer chain: (**a**) representative volume element; (**b**) point A-$r_{p}/l$ = 10, *k* = 1; (**c**) point A-$r_{p}/l$ = 10, *k* = 10; (**d**) point A-$r_{p}/l$ = 100, *k* = 1; (**e**) point B-$r_{p}/l$ = 10, *k* = 1; (**f**) point C-$r_{p}/l$ = 10, *k* = 1.

In this section, the local distribution of polymer chains is investigated, providing a basis for the next section. Particles with radius *r_p_* are regarded as homogeneous distribution inside the matrix ideally, as the representative volume element (length *d*) shown in Figure 13a. The advantage of this assumption is that the computational time can be saved rather than considering the real microstructure of the particle reinforced polymer. From Figure 13b–d, the effect of volume fraction *v* = $4\pi r_{p}^{3}/\left(3d^{3}\right)$, segment number *n*, particle radius-to-segment length ratio $r_{p}/l$, and parameter *k* on the local density (P) was studied and compared with that of random distribution (P_rand_). The calculation results show that local density increased with increasing *k* and decreasing $r_{p}/l$. From Figure 13a,e,f, the local densities of different positions (points A, B and C) were calculated. The calculation results indicate that local density was larger when the point was closer to the boundary. Beyond that, local density increased with segment number *n* and volume fraction *v*, as shown in Figure 13.

### 4.2. Simulation of the Physical Parameters

**Figure 14:**
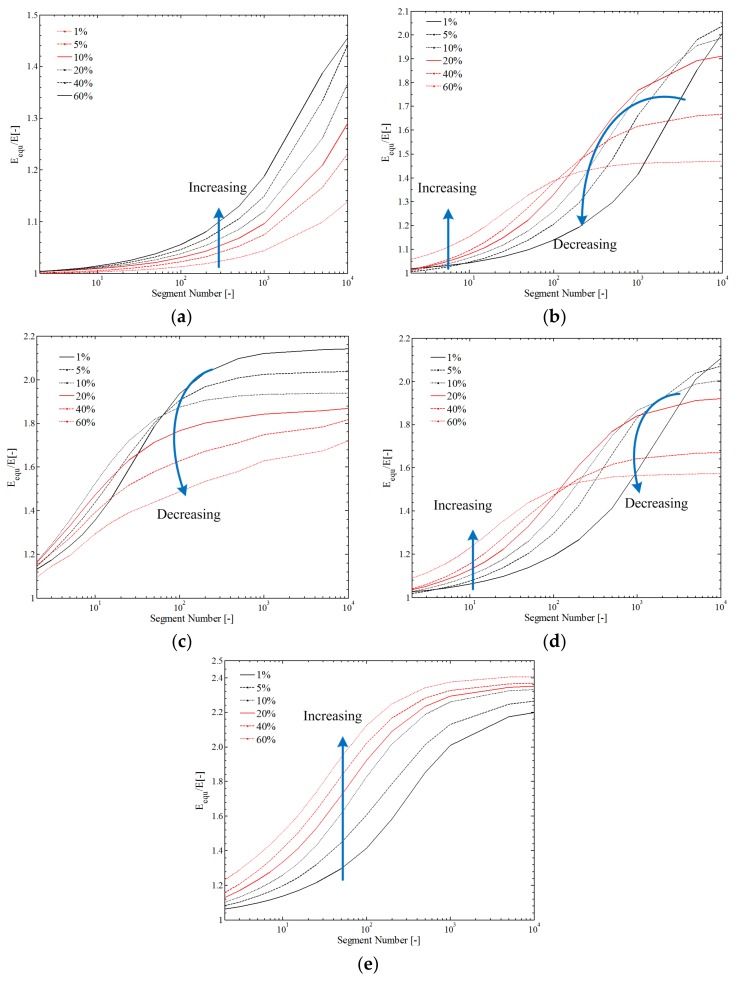
The effect of internal geometry on the equivalent elastic modulus: (**a**) $r_{p}/l$ = 100; *k* = 1; (**b**) $r_{p}/l$ = 10; *k* = 1; (**c**) $r_{p}/l$ = 1; *k* = 1; (**d**) $r_{p}/l$ = 10; *k* = 2; (**e**) $r_{p}/l$ = 10; *k* = 10.

The effects of internal particles on material electromechanical properties are mainly discussed in this section. The particles (CCTO) were regarded as homogeneously distributed inside the material. With different particle volume fractions, the reinforcements of the particles are plotted in Figure 14, from which the equivalent elastic modulus can be observed as a function of *l*, *n*, and *k*.

As can be seen in Figure 14, the equivalent elastic modulus generally increased with increasing segment number *n*. With a large particle radius-to-segment length ratio ($r_{p}/l$ = 100), the equivalent elastic modulus increased with the volume fraction, as shown in Figure 14a. Differently, with a small particle radius-to-segment length ratio ($r_{p}/l$ = 10 and $r_{p}/l$ = 1), the equivalent elastic modulus increased with the volume fraction over a range of segment numbers and then decreased, as shown in Figure 14b,c. When comparing Figure 14b,d, the reinforcement of cross-linking characteristic can be observed: the equivalent elastic modulus was generally enhanced (about 10%). In Figure 14e, the reinforcement is more obvious than that of *k* = 2:70% for volume fraction 0.6; 10% for volume fraction 0.05.

**Figure 15:**
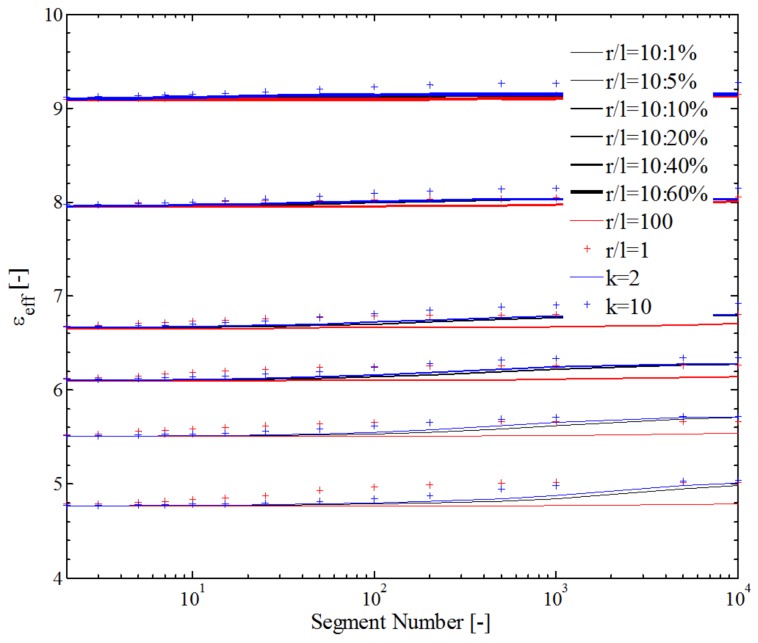
Effect of internal filler on the dielectric permittivity of polymer-based composite.

In addition, the effect of internal particles on the dielectric property was evaluated. According to the research on CCTO–PDMS composites, the dielectric permittivity for CCTO after sintering was almost 10^5^ under a frequency of 10^5^ Hz; the dielectric permittivity for CCTO after the grounding process was 10^2^–10^3^ under frequency < 10^2^ Hz (chosen as $\varepsilon_{c}=142.92$); for PDMS, the dielectric permittivity almost held constant (chosen as $\varepsilon_{p}=3.16$) over a broad frequency range (10^−1^–10^6^ Hz). In contrast to the equivalent elastic modulus mentioned above, the dielectric permittivity was mainly affected by volume fraction, although there was a small increase in $\varepsilon_{\mathrm{eff}}$ at *n* = 10^6^ compared to that at *n* = 1–10 (about 10%), as shown in Figure 15. The main reason for the difference is the colossal dielectric permittivity of CCTO.

### 4.3. Simulation of CCTO-PDMS

**Figure 16:**
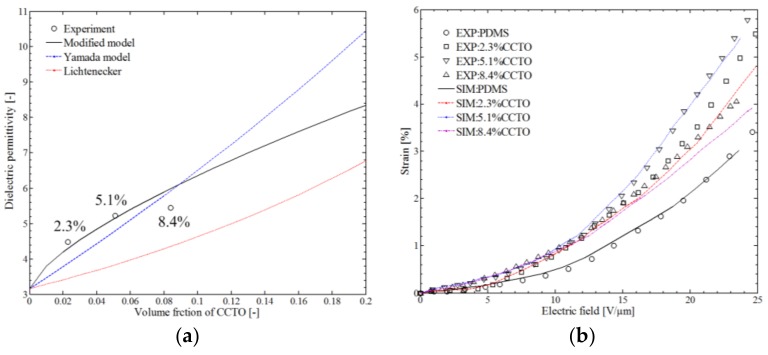
Simulation results of the CaCu3Ti4O12 (CCTO)–PDMS: (**a**) dielectric permittivity; (**b**) electromechanical behavior [25].

The details of the fabrication and the test process of CCTO-PDMS with different volume fractions of CCTO (2.3%, 5.1%, and 8.4%) can be found in the literature [25]. In this section, the parameters $r_{p}/l$ = 25, *n* = 42, and *k* = 2 were employed in the simulation. For comparison purposes, the calculation results of Lichtenecker’s logarithmic law and Yamada’s model of mixing of composites are added in Figure 16a. Using the same parameters, the electromechanical behavior of CCTO-PDMS was also calculated, as shown in Figure 16b. The Lichtenecker logarithmic law and Yamada’s model can be expressed as [25,50]:

$$\text{Lichtenecker:}\;\log\left(\varepsilon_{eff}\right)=v_{f}\log\left(\varepsilon_{f}\right)+v_{m}\log\left(\varepsilon_{m}\right),$$

(21) $$\text{Yamada:}\;\varepsilon_{eff}=\varepsilon_{m}\left[1+\frac{n_{Y}v_{f}\left(\varepsilon_{f}-\varepsilon_{m}\right)}{n_{Y}\varepsilon_{m}+\left(\varepsilon_{f}-\varepsilon_{m}\right)v_{m}}\right].$$

## 5. Conclusions

In summary, we have proposed a numerical method to capture the electromechanical behavior of polymer-based composites. The distribution of polymer chains was modified considering several factors: segment number, segment length, external geometries, and internal fillers. Generally, inside the system the chains were regarded as random distribution; however, near any surfaces (inside or outside), the distribution of the chains was restricted. The advantage of the model is that many physical parameters related to local polymer chain can be investigated.

As for VHB 4910 film, it is suggested that the polymer chain is restricted along the thickness direction. The simulation results show that when the deformation increased due to electromechanical coupling, the activated part had the potential to increase the instability and further deform owing to the lower corresponding elastic modulus. More importantly, the electromechanical coupling effect was increased by this phenomenon compared with the isotropic assumption. For a complex system, it seems that the electric field is a function of the surface characteristics, which change during the loading process.

For the CCTO–PDMS composite, the calculation results indicate that the electromechanical properties are greatly affected by internal fillers. With a higher density of CCTO, a higher elastic modulus and dielectric was generally found. Moreover, interaction between the filler and the matrix also contributes to the reinforcement, up to a critical level. The proposed model was successfully used to estimate the experimental data in the literature, and can promote the design and development of composite materials.

## Supplementary Materials

The following are available online at http://www.mdpi.com/2073-4360/10/4/426/s1, Table S1: Material parameters.
