# Supplementary material for: A Numerical Method Charactering the Electromechanical Properties of Particle Reinforced Composite Based on Statistics
Source: Polymers (Basel). 2018 Apr 11;10(4):426. doi: 10.3390/polym10040426 (PMC6415452; doi:10.3390/polym10040426)
Supplement: Supplementary file 1 [file polymers-10-00426-s001.pdf]

Table S1. Material parameters.

Modified model	$E_{ser,1}$ N/mm^2	$E_{par,1}$ N/mm^2	$\eta_{par,1}$ N/mm^2	$E_{ser,2}$ N/mm^2	$E_{par,2}$ N/mm^2	$\eta_{par,2}$ N/mm^2	$E_{ser,3}$ N/mm^2	$E_{par,3}$ N/mm^2	$\eta_{par,3}$ N/mm^2	l mm	N -
	0.0473	0.0864	32.17	0.036	-	0.568	0.037	-	0.061	0.08	15
Yeoh model	g_1	t_1(s)	g_2	t_2(s)	g_3	t_3(s)	g_4	t_4(s)	C_1 MPA	C_2 MPA	C_3 MPA
	0.478	0.153	0.205	0.464	0.0072	32.02	0.0492	215.8	8.27×10^{-2}	-7.5×10^{-4}	5.86×10^{-6}
VHB 4910	ε -	ε_0 As/Vm	a -	b -	c -						
	4.7	8.5×10^{-12}	-2.9×10^{-1}	9.0×10^{-2}	-9.8×10^{-3}						
